# Cross-Reactivity of Virus-Specific CD8+ T Cells Against Allogeneic HLA-C: Possible Implications for Pregnancy Outcome

**DOI:** 10.3389/fimmu.2018.02880

**Published:** 2018-12-06

**Authors:** Anita van der Zwan, Ellen M. W. van der Meer-Prins, Paula P. M. C. van Miert, Heleen van den Heuvel, Jacqueline D. H. Anholts, Dave L. Roelen, Frans H. J. Claas, Sebastiaan Heidt

**Affiliations:** Department of Immunohematology and Blood Transfusion, Leiden University Medical Center, Leiden, Netherlands

**Keywords:** heterologous immunity, virus-specific T cells, allogeneic HLA, HLA-C, pregnancy

## Abstract

Heterologous immunity of virus-specific T cells poses a potential barrier to transplantation tolerance. Cross-reactivity to HLA-A and -B molecules has broadly been described, whereas responses to allo-HLA-C have remained ill defined. In contrast to the transplant setting, HLA-C is the only polymorphic HLA molecule expressed by extravillous trophoblasts at the maternal-fetal interface during pregnancy. Uncontrolled placental viral infections, accompanied by a pro-inflammatory milieu, can alter the activation status and stability of effector T cells. Potential cross-reactivity of maternal decidual virus-specific T cells to fetal allo-HLA-C may thereby have detrimental consequences for the success of pregnancy. To explore the presence of cross-reactivity to HLA-C and the other non-classical HLA antigens expressed by trophoblasts, HLA-A and -B-restricted CD8+ T cells specific for Epstein-Barr virus, Cytomegalovirus, Varicella-Zoster virus, and Influenza virus were tested against target cells expressing HLA-C, -E, and -G molecules. An HLA-B^*^08:01-restricted EBV-specific T cell clone displayed cross-reactivity against HLA-C^*^01:02. Furthermore, cross-reactivity of HLA-C-restricted virus-specific CD8+ T cells was observed for HCMV HLA-C^*^06:02/TRA CD8+ T cell lines and clones against HLA-C^*^03:02. Collectively, these results demonstrate that cross-reactivity against HLA-C can occur and thereby may affect pregnancy outcome.

## 1. Introduction

High frequencies of memory T cells against several viruses such as Influenza virus (FLU), Epstein-Barr virus (EBV), Human Cytomegalovirus (HCMV), and Varicella-Zoster virus (VZV) have been described in healthy individuals (1–3). Primary infection or reactivation of these viruses can compromise graft survival after transplantation and during pregnancy result in fetal malformation and pregnancy complications such as preterm birth and intrauterine growth restriction (4–7). A significant proportion of virus-specific CD8+ T cells in healthy (non-HLA sensitized) individuals display alloreactivity against allogeneic human leukocyte antigens (allo-HLA) (8, 9). This phenomenon, referred to as heterologous immunity, enables the same T-cell receptor (TCR) to recognize its autologous virus-peptide presenting HLA allele as well as allo-HLA. The avidity of these cross-reactive T cells for their viral and allogeneic targets depends on the levels of peptide presentation (10). Within an individual, an HLA-restricted virus-specific T cell response can generate several clonotypes that have different patterns of allo-HLA cross-reactivity (11). Both naïve and memory T cells show alloreactive potential, though memory T cells pose a superior threat (12, 13). Their activation threshold is significantly lower as they have less need for co-stimulation while their cytotoxic function is enhanced (14, 15). To ensure a comprehensive immune response to foreign antigens, this high degree of cross-reactivity is an intrinsic and essential feature of antigen recognition by T cells, of which allo-HLA cross-reactivity is an inherent consequence (16).

In the pregnancy setting T cells have a dual role in mediating tolerance toward the allogeneic fetus and at the same time responding to infections. During gestation, fetal extravillous trophoblasts (EVT) deeply invade the maternal tissues (decidua) where they establish direct contact with the maternal immune cells. EVT do not express the highly polymorphic HLA-A and -B, but do express HLA-C, -E, and -G (17, 18). CD8+ T cells present in the decidua demonstrate a mixed transcriptional profile of T cell dysfunction, activation and effector function. They are not permanently suppressed, but maintain the capacity to respond to proinflammatory occurrences, such as infections (19). Significant numbers of HLA-A and -B-restricted virus-specific CD8+ T cells are found in decidual tissue of term pregnancy (20). Furthermore, in the maternal peripheral blood, cytotoxic T lymphocyte (CTL) responses to paternal allo-HLA (HLA-A/B) and minor histocompatibility antigens (mHag) have been detected during pregnancy (21–23). Thus, maternal CD8+ T cells can respond to viral, fetal and placental antigens during pregnancy but so far no evidence exists on the presence of HLA-C-restricted viral and mHag-specific CD8+ T cells and whether maternal HLA-A and -B-restricted virus-specific CD8+ T cells can cross-react with fetal HLA antigens, leading to possible pregnancy complications. Recognition of fetal HLA-C by both B cells and helper T cells is suggested by the presence of specific HLA-C IgG antibodies in women with recurrent miscarriages (24). Furthermore, HLA-C incompatibility is significantly increased in couples with unexplained recurrent miscarriages when compared to control subjects (25). In addition, certain combinations of maternal killer cell immunoglobulin-like receptor (KIR) genotypes, expressed by decidual NK cells, and fetal HLA-C are associated with pregnancy complications such as preeclampsia (26). These data indicate that fetal HLA-C could play a vital role in guiding the maternal immune response during pregnancy.

Studies on heterologous immunity in transplantation have focused on the cross-reactivity of HLA-A and -B-restricted virus-specific CD8+ T cells with allogeneic HLA-A and -B, with less attention for HLA-C considering its lower cell surface expression levels when compared to HLA-A and -B (27). In the context of pregnancy HLA-C is the only polymorphic antigen expressed on EVT and alloreactivity to HLA-C (and HLA-E and -G) is therefore unique and highly significant. The importance of HLA-C incompatibility in pregnancy complications coupled to the presence of virus-specific CD8+ T cells at the maternal-fetal interface, led us to investigate whether cross-reactivity of virus-specific CD8+ T cells against HLA-C, -E and -G is a common phenomenon in healthy individuals.

## 2. Results

### 2.1. Alloreactivity of an EBV B8/FLR CD8+ T cell Clone 4D5 Against HLA-C^*^01:02

**Figure 1:**
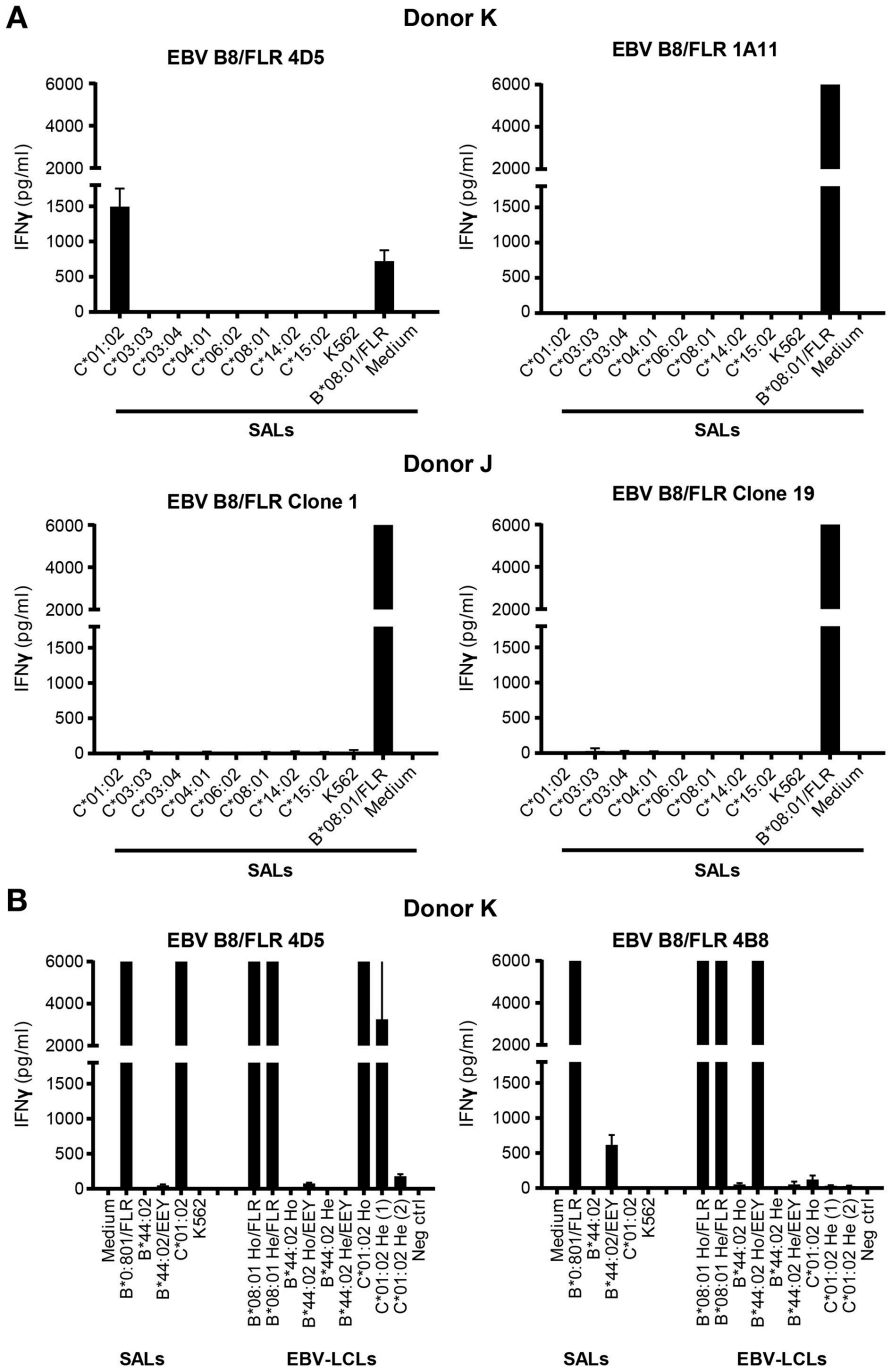
Alloreactivity of EBV B8/FLR T cell clone 4D5 against HLA-C^*^01:02. **(A)** EBV B8/FLR T cell lines (*n* = 9; 1A11 shown) and T cell clones (*n* = 6; 4D5, clone 1, and clone 19 shown) were stimulated with a panel of HLA-C expressing SALs after which IFNγ production was measured. EBV B8/FLR T cell clone 4D5 showed alloreactivity against HLA-C^*^01:02. **(B)** One EBV B8/FLR T cell line and four EBV B8/FLR T cell clones (4B8 and 4D5 shown) were stimulated with a panel of SALs and EBV-LCLs expressing HLA-B^*^08:01, HLA-C^*^01:02, and HLA-B^*^44:02 alleles after which IFNγ production was measured. The range of the ELISA standard curve: 5–5120 pg/ml; Ho, homozygous; He, heterozygous. Bars represent duplicate values with standard deviation of the mean.

To investigate the ability of virus-specific CD8+ T cells to cross-react with HLA-C, -E, and -G, 29 HLA-A and -B-restricted human CMV, FLU, VZV, and EBV-specific CD8+ T cell lines and clones (28) were tested against a panel of single antigen expressing lines (SALs) expressing HLA-C, -E, and -G alleles (*n* = 11) (29, 30). An HLA-A2-restricted EBV-specific CD8+ T cell clone isolated from placental decidua parietalis was also included (20). The specificities of the isolated virus-specific CD8+ T cell lines and clones are listed in Table 1. Lack of IFNγ production revealed that alloreactivity against HLA-C, -E, and -G is not common Table 2. Nonetheless, one HLA-B^*^08:01-restricted EBV-specific (EBV B8/FLR) T cell clone, 4D5, showed significant alloreactivity against HLA-C^*^01:02 Figure 1A. This T cell clone was isolated from an HLA-C^*^01:02 negative donor.

**Table 1:** Specificities of isolated virus-specific CD8+ T cell lines and clones.

**Virus**	**HLA**	**Antigen**	**Epitope**
HCMV	HLA-A^*^02:01	pp65 (495-503)	NLVPMVATV
HCMV	HLA-B^*^35:01	pp65 (123-131)	IPSINVHHY
HCMV	HLA-C^*^06:02	pp65 (201-209)	TRATKMQVI
HCMV	HLA-C^*^07:02	IE-1 (309-317)	CRVLCCYVL
EBV	HLA-A^*^02:01	BMLF1 (280-288)	GLCTLVAML
EBV	HLA-B^*^08:01	EBNA-3A (325-333)	FLRGRAYGL
EBV	HLA-B^*^35:01	EBNA-3A (458-466)	YPLHEQHGM
FLU	HLA-A^*^02:01	IMP (58-66)	GILGFVFTL
VZV	HLA-A^*^02:01	IE-62 (593-601)	ALWALPHAA

*HCMV, human Cytomegalovirus; EBV, Epstein-Barr virus; FLU, Influenza virus; VZV, Varicella-Zoster virus*.

**Table 2:** Alloreactivity of virus-specific CD8+ T cell lines and clones against HLA-C, -E, and -G.

**Donor**	**Specificity**	**TCR V*β***	**# T cell** **lines/clones tested**	**Allo-HLA-C**	**Allo-HLA-E, -G**	**Target cell (s)**	**Allo-HLA-C, -E,** **-G cross-reactivity**
A	HCMV A2/NLV	2	2	2	2	SALs	No
	HCMV A2/NLV	8	3	2	3	SALs	No
	HCMV A2/NLV	13.1	1	0	1	SALs	No
	HCMV A2/NLV	5.2	1	0	1	SALs	No
	HCMV A2/NLV	#	9	0	9	SALs	No
A	HCMV B35/IPS	2	1	0	1	SALs	No
	HCMV B35/IPS	3	2	2	2	SALs	No
	HCMV B35/IPS	13.2	2	1	2	SALs	No
	HCMV B35/IPS	22	1	1	1	SALs	No
	HCMV B35/IPS	^*^	2	2	2	SALs	No
B	HCMV B35/IPS	3	3	2	3	SALs	No
C	HCMV B35/IPS	3	4	2	4	SALs	No
A	FLU A2/GIL	17	3	2	3	SALs	No
	FLU A2/GIL	17+18	1	0	1	SALs	No
D	FLU A2/GIL	17	4	1	4	SALs	No
B	FLU A2/GIL	#	3	2	3	SALs	No
E	FLU A2/GIL	#	2	1	2	SALs	No
F	FLU A2/GIL	#	1	0	1	SALs	No
F	VZV A2/ALW	#	1	1	1	SALs	No
G	VZV A2/ALW	#	1	1	1	SALs	No
H	EBV B35/YPL	14	1	1	1	SALs	No
	EBV B35/YPL	21.3	2	2	2	SALs	No
	EBV B35/YPL	^*^	1	1	1	SALs	No
I	EBV A2/GLC (decidua parietalis)	#	1	1	1	SALs	No
J	EBV B8/FLR	7	2	2	2	SALs, EBV-LCLs	No
K	EBV B8/FLR (4D5)	3	1	1	1	SALs, EBV-LCLs, PHA blasts	HLA-C^*^01:02
	EBV B8/FLR	4	2	2	2	SALs, EBV-LCLs	No
L	HCMV C^*^0702/CRV	6	1	1	1	721.221, EBV-LCLs	No
M	HCMV C^*^0602/TRA	13	2	2	2	SALs, EBV-LCLs	No
	HCMV C^*^0602/TRA (1A3, 7A12, 10C1)	28	2	2	2	SALs, EBV-LCLs, PHA blasts	HLA-C^*^03:02
						**Summary**	
^*^ The TCR Vβ could not be determined with the TCR Vβ kit used.						Specificities	9
# Not tested.						Donors	13
						TCR tested	21
						T cell lines/clones tested against HLA-C, -E, -G	34

To corroborate alloreactivity against HLA-C^*^01:02, one EBV B8/FLR T cell line and four T cell clones were stimulated with a panel of SALs and EBV lymphoblastoid cell lines (EBV-LCLs) expressing HLA-C^*^01:02 and HLA-B^*^44:02 alleles for 24 h after which IFNγ production was measured. Alloreactivity of EBV B8/FLR T cells against HLA-B^*^44:02 is a commonly described occurrence (31). T cell clone 4D5 reacted against its virus-specific restriction allele HLA-B^*^08:01 loaded with FLR peptide as well as HLA-C^*^01:02 expressed by SALs and EBV-LCLs. Its lower alloreactivity against the second EBV-LCL donor expressing heterozygous HLA-C^*^01:02 may have been a result of low HLA-C expression. T cell clone 4D5 did not show alloreactivity against HLA-B^*^44:02 Figure 1B. T cell clone 4B8 (here shown as a representative example), comprising a different TCR Vα and Vβ usage than 4D5 Table 3, displayed no alloreactivity against HLA-C^*^01:02 and only cross-reacted with HLA-B^*^44:02 when loaded with the appropriate self-peptide (EEY). The other EBV B8/FLR CD8+ T cells tested also did not cross-react with HLA-C^*^01:02, but displayed cross-reactivity against HLA-B^*^44:02. No alloreactivity against HLA-E and -G was discerned Figure S1.

**Table 3:** TCR Vα and Vβ usage of CD8+T cell lines and clones.

**T cell line/clone**	**TRαV**	**TRαJ**	**CDR3α**	**TRβV**	**TRβJ**	**TRβD**	**CDR3β**	**Cross-reactivity**
B8/FLR T cell clone 4B8	TRAV1-2^*^01 F	TRAJ36^*^01 F	C A V R D Q T G A N N L F F	TRBV4-3^*^02 (F)	TRBJ2-5^*^01 F	TRBD2^*^01 F	C A S S H G L A G I L E T Q Y F	No
B8/FLR T cell clone 4D5	TRAV40^*^01 F	TRAJ43^*^01 F	C L L G D N D M R F	TRBV3-1/2^*^01	TRBJ1-6^*^02 F	TRBD2^*^01 F	C A S S Q P P T G R S Y N S P L H F	HLA-C^*^01:02
C^*^0702/CRV T cell clone LH	TRAV27^*^03 (F)	TRAJ33^*^01 F	C A G G D M D S N Y Q L I W	TRBV6-2/3/6	TRBJ2-7^*^01 F	TRBD1^*^01 F	C A S G E V Y E Q Y F	No
C^*^0602/TRA T cell clone 1F12	TRAV19^*^01 F	TRAJ26^*^01 F	C A L S E G G S Y G Q N F V F	TRBV13^*^01/02 (F)	TRBJ2-1^*^01 F	TRBD2^*^01 F	C A S S L R D E Q F F	No
C^*^0602/TRA T cell line 1A3	TRAV12-2^*^02 (F)	TRAJ20^*^01 F (a)	C A V N N D Y K L S N	TRBV28^*^01 F	TRBJ2-1^*^01 F	TRBD2^*^01 F	C A S S S G G L E N E Q F F	HLA-C^*^03:02
C^*^0602/TRA T cell clone 10C1				TRBV28^*^01 F	TRBJ2-1^*^01 F	TRBD2^*^01 F	C A S S S G G L E N E Q F F	HLA-C^*^03:02

**Figure 2:**
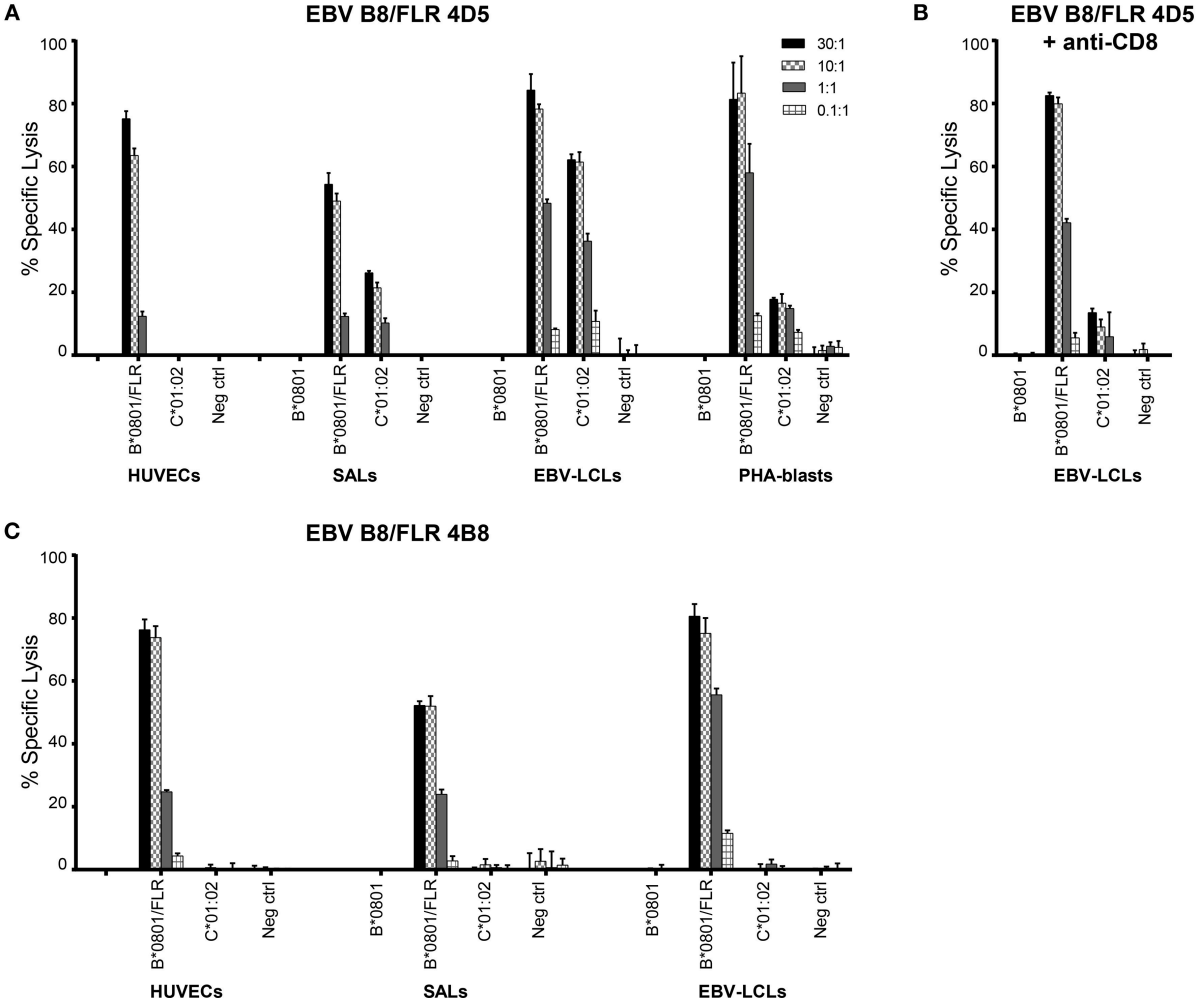
EBV B8/FLR T cell clone 4D5 is cytotoxic against HLA-C^*^01:02. Cytotoxicity of T cell clones 4D5 **(A)** and 4B8 **(C)** was tested against^51^Cr-labeled HUVECs, SALs, EBV-LCLs, and PHA blasts expressing the recognized allo-HLA-C^*^01:02 allele and the virus-specific restriction HLA allele loaded with viral peptide as a positive control. As a negative control, target cells expressing no HLA-B^*^0801 and HLA-C^*^0102 were included. **(B)** T cell clone 4D5 was incubated with an anti-CD8 blocking antibody prior to co-culture with^51^Cr-labeled EBV-LCLs and specific lysis was measured. Bars represent triplicate values with standard deviation of the mean.

Alloreactivity of virus-specific CD8+ T cells can be cell type or tissue-specific (32, 9). Therefore, to further functionally validate our results, cytotoxicity of the T cell clones 4D5 and 4B8 was investigated against^51^Chromium (^51^Cr) -labeled human umbilical vein endothelial cells (HUVECs), SALs (myeloid origin), EBV-LCLs (B cells) and PHA blasts (T cells) expressing the recognized allo-HLA-C^*^01:02 allele and the virus-specific restriction allele HLA-B^*^08:01 loaded with viral peptide as a positive control. Target cells expressing no HLA-B^*^08:01 and HLA-C^*^01:02 were included as a negative control. The T cell clones were added to their targets in four effector: target ratios. Specific lysis of HLA-C^*^01:02 expressing SALs, EBV-LCLs and PHA blasts by T cell clone 4D5 was observed in a ratio-dependent manner. T cell clone 4D5 was however not lytic against HUVECs expressing HLA-C^*^01:02, presumably the result of the relevant self-peptide not being expressed by this cell type (33) Figure 2A. The robust cytolytic response of T cell clone 4D5 against EBV-LCLs expressing HLA-C^*^01:02 was substantially decreased by addition of an anti-CD8 blocking antibody, while lysis of target cells expressing the virus-specific restriction allele HLA-B^*^08:01 loaded with FLR peptide was not affected, indicating distinct TCR affinities Figure 2B. No specific lysis by T cell clone 4B8 was observed Figure 2C. Together, these results demonstrate that alloreactivity of HLA-A and -B-restricted virus-specific CD8+ T cells against HLA-C can occur and is dependent on CD8.

### 2.2. Characterization of HLA-C^*^06:02-Restricted HCMV-Specific T Cell Lines and Clones

**Figure 3:**
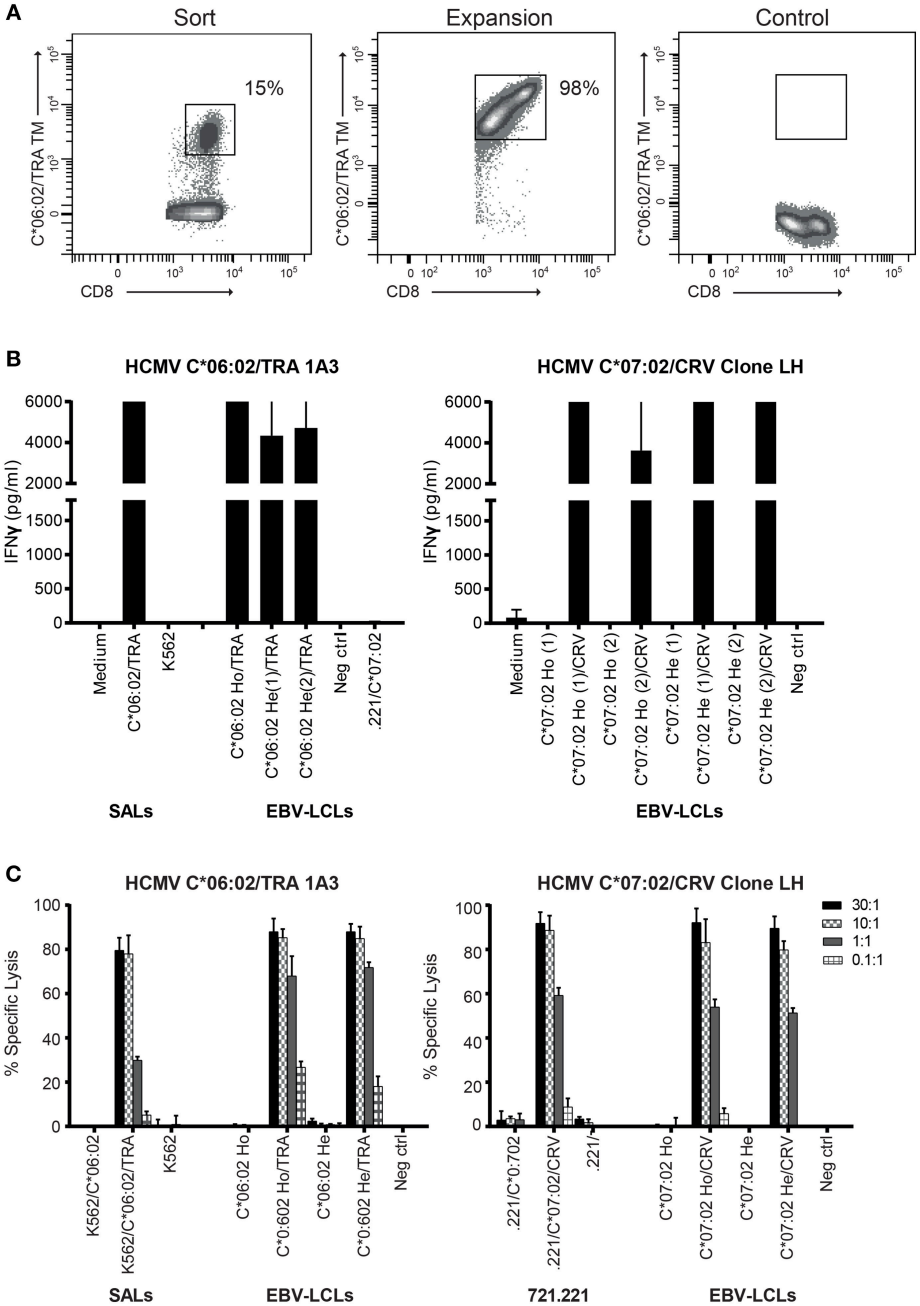
Generated HCMV HLA-C^*^06:02/TRA T cell lines and clones, and the HCMV HLA-C^*^07:02/CRV T cell clone are cytolytic against their target cells. **(A)** FACS plots of HLA-C^*^06:02/TRA tetramer staining at time of sorting and 2 weeks after expansion of the tetramer-positive CD8+ T cells. **(B)** Two HCMV C^*^06:02/TRA T cell lines (1A3 shown) and two T cell clones, and HCMV C^*^07:02/CRV T cell clone LH were stimulated with SALs and EBV-LCLs expressing HLA-C^*^06:02 or C^*^07:02 loaded with viral peptide. **(C)** Cytotoxicity of one HCMV C^*^06:02/TRA T cell line (1A3 shown) and two T cell clones, and HCMV C^*^07:02/CRV T cell clone LH was tested against^51^Cr-labeled SALs, 721.221 cells and EBV-LCLs expressing the virus-specific restriction HLA allele alone or loaded with viral peptide. The range of the ELISA standard curve: 5–5120 pg/ml. Ho, homozygous; He, heterozygous. Bars represent triplicate values with standard deviation of the mean.

HLA-C-restricted virus-specific CD8+ T cells have been described in the context of HIV infection where they recognize a highly conserved epitope and in HCMV infection where HLA-C^*^07:02-restricted CD8+ T cells dominate the T cell response to the immediate-early 1 (IE-1) viral antigen and their levels increase with age (34–36). Given their high allele frequency in the population, we set out to isolate HLA-C^*^06:02- and HLA-C^*^07:02-restricted HCMV-specific CD8+ T cells (37, 38) and explore their alloreactivity against HLA-C, -E, and -G. PBMC of HLA-C^*^06:02+HCMV+ donors (*n* = 10) were stained with an HLA-C^*^06:02 tetramer containing the HCMV TRA peptide (39) Table 1. From a donor with 15% positivity for the HLA-C^*^06:02/TRA tetramer, CD8+ T cell lines and clones were generated by sorting tetramer positive CD8+ T cells and expanding them *in vitro* Figure 3A; Figure S2. An established HLA-C^*^07:02-restricted HCMV-specific CD8+ T cell clone (LH) was included in the analysis (35). To examine the functionality of these HLA-C^*^06:02/TRA-restricted HCMV-specific T cell lines and clones, as well as the HLA-C^*^07:02/CRV-restricted HCMV-specific T cell clone LH, IFNγ production was measured after 24 h of co-culture with SALs and EBV-LCLs expressing HLA-C^*^06:02 or C^*^07:02 loaded with the appropriate viral peptide. All HLA-C^*^06:02-restricted T cell lines and clones, and the HLA-C^*^07:02-restricted clone LH responded against their virus-specific restriction HLA-allele loaded with viral peptide Figure 3B. In addition, specific lysis in a ratio-dependent manner of^51^Cr-labeled SALs, 721.221 cells expressing HLA-C^*^07:02, and EBV-LCLs was detected Figure 3C. These results confirmed functionality of the generated HLA-C^*^06:02-restricted T cell lines and clones (HCMV C^*^06:02/TRA), and the established HLA-C^*^07:02-restricted T cell clone LH (HCMV C^*^07:02/CRV).

### 2.3. Alloreactivity and Cytotoxicity of HCMV C^*^06:02/TRA T Cell Lines Against HLA-C^*^03:02

**Figure 4:**
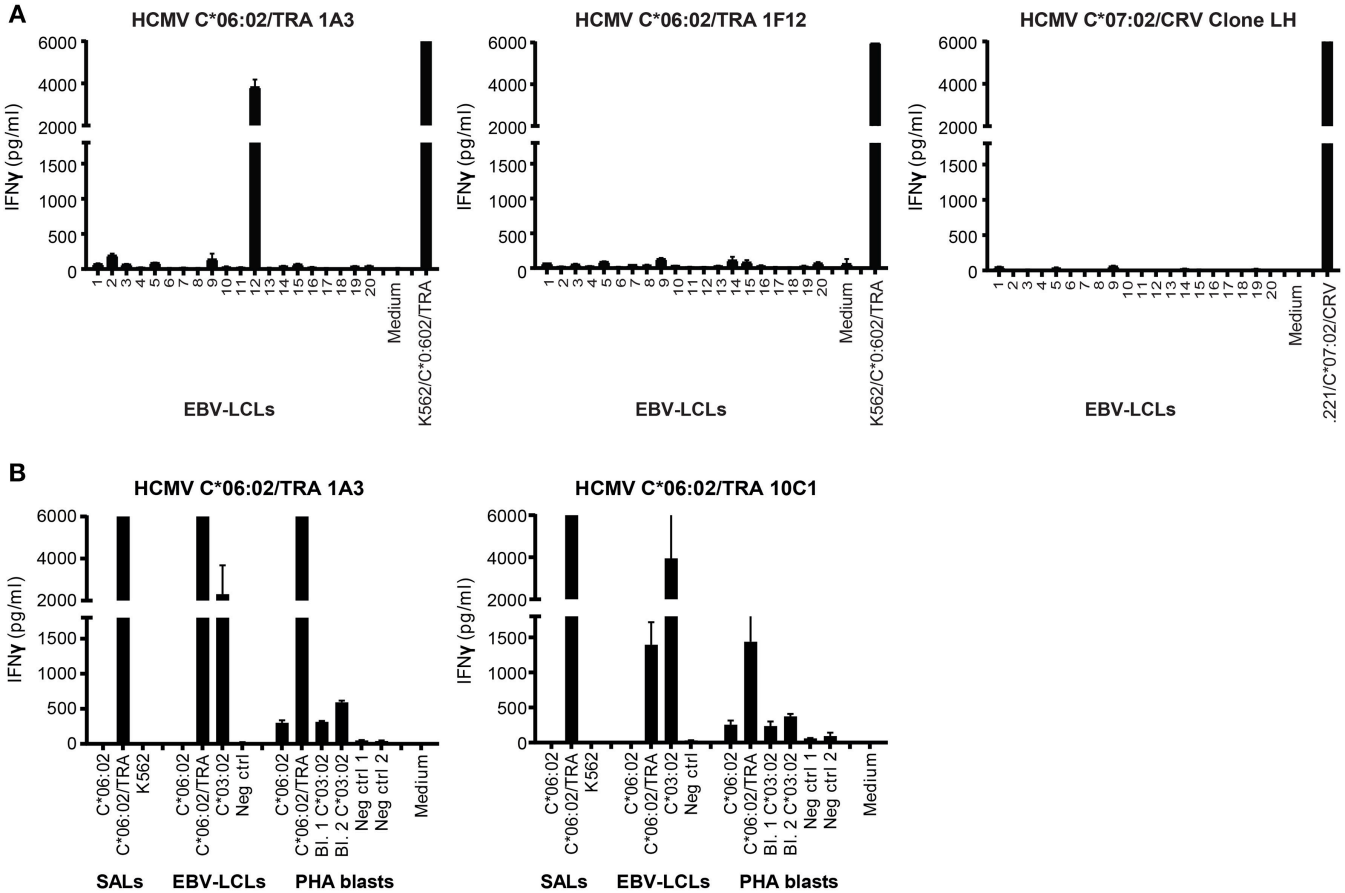
Alloreactivity of HCMV C^*^06:02/TRA T cell lines against HLA-C^*^03:02. **(A)** Two HCMV C^*^06:02/TRA T cell lines (1A3 shown) and two T cell clones (1F12 shown), and HCMV C^*^07:02/CRV T cell clone LH were stimulated with a panel of EBV-LCLs after which IFNγ production was measured. **(B)** Three HCMV C^*^06:02/TRA T cell lines (1A3 shown) and five T cell clones (10C1 shown) were stimulated with EBV-LCLs and PHA blasts, obtained from two different donors, expressing the recognized allo-HLA-C^*^03:02 allele and the virus-specific restriction allele HLA-C^*^06:02 loaded with viral peptide as a positive control. SALs expressing HLA-C^*^06:02 loaded with viral peptide were also included as a positive control. The range of the ELISA standard curve: 5–5120 pg/ml. Bars represent duplicate values with standard deviation of the mean.

Next, the HCMV C^*^06:02/TRA CD8+ T lines and clones (*n* = 4), and HCMV C^*^07:02/CRV CD8+ T cell clone LH were tested against a panel of SALs expressing HLA-C, -E, and -G, in a co-culture system where IFNγ production was assessed. No alloreactivity was observed in this setting Figures S3A,B. Subsequently, in a similar manner, alloreactivity against a panel of EBV-LCLs covering the most common HLA alleles was investigated Table 4. Interestingly, two HCMV C^*^06:02/TRA T cell lines cross-reacted with EBV-LCL donor 12 of which T cell line 1A3 is shown as a representative example Figure 4A. When comparing the HLA typing of all 20 EBV-LCL donors in the panel, HLA-C^*^03:02 expressed by donor 12 was the only non-overlapping HLA allele candidate. A SAL expressing HLA-C^*^03:02 is not present in the panel and therefore cross-reactivity against this allele was not picked up in the initial screening Figures 3SA,B. A role for HLA class II was ruled out. CD8+ T cell lines and clones cross-reacting against donor 12 disclosed a distinct TCR Vα and Vβ usage when compared to CD8+ T cells showing no alloreactivity Table 3. No alloreactivity of the HCMV C^*^07:02/CRV clone LH was observed Figure 4A.

**Table 4:** HLA class I typing of the EBV-LCL panel.

	**HLA class I**		
**Panel ID**	**A**	**B**	**C**
1	02:01, 32:01	08:01, 44:05	02:02, 07:01/06/18
2	24:02, 33:01	14:02	02:02/32, 08:02/29
3	03:01/22, 29:02/09	07:02/61/114, 44:03/105	07:02, 16:01
4	11:01/43, 33:03/51	18:01/17N, 52:01	07:02, 07:04
5	11:01/33, 30:01/54	13:02, 35:01/42	04:01, 06:02
6	01:01, 26:01	08:01, 49:01	07:01
7	11:01, 31:01	15:01, 57:01	03:03, 06:02/55
8	02:03, 24:02	38:02, 40:01	03:04, 07:02
9	29:02, 31:01	18:01/17N, 58:01	05:01, 07:18/01
10	24:03	51:01	15:02
11	26:01	38:01	12:03
12	24:02, 30:01	51:01, 58:01	01:02, 03:02
13	02:01, 03:01	15:01	03:03
14	68:01, 68:02	44:02/19N, 55:01	03:03, 07:04
15	24:02, 31:01	39:01, 55:01	03:03, 12:03
16	30:01, 68:02	42:01	17:01
17	01:01	41:01	17:01
18	02:01, 11:01	35:01, 44:03	04:01, 16:01
19	02:01, 25:01	18:01/17N/43, 44:02/19N/55	05:01, 12:03
20	24:02, 31:01/119	07:02/294/298, 07:05	07:02, 15:05

To further gauge the alloreactivity against HLA-C^*^03:02, HCMV C^*^06:02/TRA T cell lines (*n* = 2) and clones (*n* = 5) with the same TCR Vβ usage as the two CD8+ T cell lines that cross-reacted with cells from donor 12 (and all isolated from an HLA-C^*^03:02 negative donor) were stimulated with EBV-LCLs and PHA blasts expressing the recognized allo-HLA-C^*^03:02 allele. Target cells expressing the virus-specific restriction allele HLA-C^*^06:02 loaded with viral peptide were included as a positive control. Alloreactivity against HLA-C^*^03:02 was detected for all HCMV C^*^06:02 T cells tested, with substantially more IFNγ production against EBV-LCLs than against PHA blasts 1 and 2, obtained from two different donors Figure 4B. Differential HLA expression levels on the cell surface may explain increased alloreactivity against PHA blast 3 expressing HLA-C^*^03:02, obtained from a third donor Figure S4.

**Figure 5:**
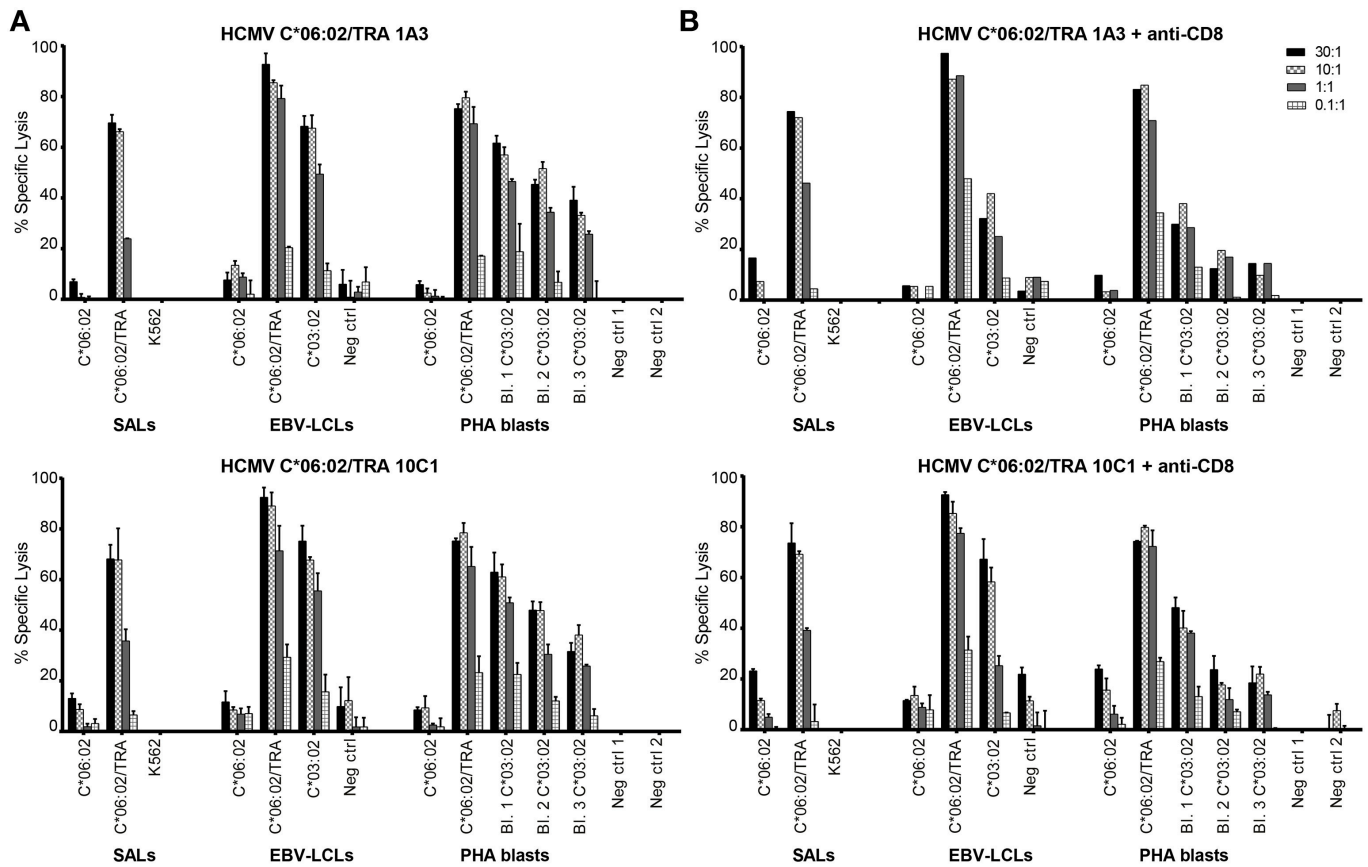
HCMV C^*^06:02/TRA T cell lines and clones are cytotoxic against HLA-C^*^03:02. **(A)** Cytotoxicity of two HCMV C^*^06:02/TRA CD8+ T cell lines (1A3 shown) and one CD8+ T cell clone (10C1) was tested against^51^Cr-labeled EBV-LCLs and PHA blasts, obtained from three different donors, expressing the recognized allo-HLA-C^*^03:02 allele and the virus-specific restriction HLA allele loaded with viral peptide as a positive control. SALs expressing HLA-C^*^06:02 loaded with viral peptide were also included as a positive control. **(B)** The CD8+ T cell lines and clone were incubated with an anti-CD8 blocking antibody prior to co-culture with target cells and specific lysis was measured. Bars represent triplicate values with standard deviation of the mean.

Having identified IFNγ production against allo-HLA-C^*^03:02, HCMV C^*^06:02/TRA T cell lines and clones were tested for cytotoxicity against^51^Cr-labeled SALs, EBV-LCLs, and PHA blasts expressing HLA-C^*^03:02. Target cells expressing the virus-specific restriction allele HLA-C^*^06:02 loaded with viral peptide were included as a positive control. The CD8+ T cell lines and clone were added to their targets in four effector: target ratios. Specific lysis of HLA-C^*^03:02 expressing target cells was observed in a ratio-dependent manner Figure 5A. Subsequently, the T cell lines and clone were incubated with an anti-CD8 blocking antibody prior to co-culture with the^51^Cr-labeled target cells resulting in substantially decreased lysis of target cells expressing HLA-C^*^03:02. A decrease in lysis was not observed for target cells expressing the virus-specific restriction allele HLA-C^*^06:02 loaded with viral peptide, indicating distinct TCR affinities Figure 5B. These findings highlight the functionality of the isolated HCMV C^*^06:02/TRA T cell lines and clones and provide evidence that alloreactivity of HLA-C-restricted virus-specific CD8+ T cells against HLA-C is a phenomenon that occurs.

## 3. Discussion

Alloreactivity of HLA-A and -B-restricted virus-specific CD8+ T cells against HLA-A and -B is common. Eighty percent of virus-specific T cell lines and 45% of virus-specific T cell clones disclosed cross-reactivity against allo-HLA molecules (9). Here, we have shown that alloreactivity of HLA-A and -B-restricted virus-specific CD8+ T cells against HLA-C can also occur. Amongst the 29 HLA-A and -B-restricted virus-specific T cells tested, one EBV B8/FLR CD8+ T cell clone 4D5 with distinct TCR Vα and Vβ usage displayed cross-reactivity and cytotoxicity against target cells expressing HLA-C^*^01:02, indicative of a more than 10 times lower frequency within the pool of HLA-A and -B-restricted virus-specific CD8+ T cells tested. This T cell clone did not reveal the classical described cross-reaction against HLA-B^*^44:02. Our preliminary data suggests that HLA-C cross-reactivity in HLA-C-restricted virus-specific T cells is more common. HLA-C^*^06:02-restricted HCMV-specific CD8+ T cells were successfully isolated from an HCMV+ donor by means of HLA-C tetramers and deemed fully functional *in vitro*. Alloreactivity of these HCMV C^*^06:02/CRV T cell lines and clones, with distinct TCR Vα and Vβ usage, was observed against HLA-C^*^03:02. This alloreactivity was mediated by IFNγ production and cytotoxicity. Viral specificity and alloreactivity are thought to be mediated by the same TCR (9), where in our setting anti-viral reactivity occurred independent of CD8, while allo-HLA-C reactivity was CD8 dependent. Differential recognition of HLA-C on SALs, EBV-LCLs, PHA blasts, and HUVECs, that did not provoke any alloreactivity, is an indication that cross-reactivity is determined by endogenous peptide (11) and supports the anticipation that tissue-specific peptides are presented and recognized. The nature of the endogenous peptide presented in HLA-C^*^01:02 and HLA-C^*^03:02, provoking the allo-response, is however unknown. Alternatively, expression of costimulatory and coinhibitory molecules by virus-specific T cells may have an influence on T cell signaling and thereby the extent of the allo-response (40). No alloreactivity against HLA-E and -G was observed. Our initial screening was against a panel of SALs expressing most, but not all HLA-C, -E, and -G molecules and we therefore may have underestimated the allo-response of virus-specific CD8+ T cells against these HLA alleles.

Variation of HLA-C expression at the cell surface can be a result of microRNA binding and discrepancies in exons that influence the structure of the peptide-binding cleft and the diversity of peptides bound by HLA-C molecules (27, 41). Differential expression of HLA-C has an influence on the ability of CD8+ T cells to mount an immune response. High expression of HLA-C has been associated with protection against infections, yet at the same time correlates with autoimmune disease (42). Nevertheless, when viral peptides are presented in the HLA-C locus, immune responses are still lower than to those presented in the HLA-A and -B loci (43). The lower expression of HLA-C at the cell surface and the differential immune responses that it triggers when compared to HLA-A and -B (44) may be an explanation for less frequent alloreactivity against HLA-C.

We speculate that alloreactivity of virus-specific CD8+ T cells against HLA-C may play a role in pregnancy complications where HLA-C is the only polymorphic HLA allele expressed by EVT. Viral infections of the fetus or the placenta can lead to severe birth defects or pregnancy loss (5). Viruses are capable of downregulating surface HLA-A and -B expression upon infection, while HLA-C expression is spared (45). EVT also persistently express HLA-C when infected with HCMV (46). HLA-A and -B-restricted virus-specific CD8+ T cells are present at the maternal-fetal interface (20) and may be capable of cross-reacting with HLA-C under certain pro-inflammatory environmental circumstances and depending on (allo) peptide expression, thereby jeopardizing the success of pregnancy. It is yet to be established whether HLA-C-restricted virus-specific CD8+ T cells are present at the maternal-fetal interface and if so, whether they are capable of mounting an immune response. While the presence of anti-HLA-C IgG antibodies has been described in women with recurrent miscarriages, the competency of virus-specific CD8+ T cells to cross-react with HLA-C raises the question whether allo-HLA-C IgG antibodies are the only player in recurrent miscarriages or whether decidual virus-specific CD8+ T cells with cross-reactive potential also play their part in pregnancy complications.

Cross-reactivity of virus-specific CD8+ T cells against HLA-C can occur and consequently our results lay the foundation for further investigation into this cross-reactivity in the context of pregnancy. Future research will focus on isolating virus-specific CD8+ T cells from the peripheral blood of women with either a healthy pregnancy or recurrent miscarriage. Alloreactivity and differences thereof by these virus-specific CD8+ T cells, obtained after normal pregnancy and miscarriage cases, against target cells expressing allo-HLA-C, -E, and -G molecules can then be investigated. Isolating viable HLA-G+ EVT from first trimester and term placentas that express the correct HLA typing is challenging. Yet, it is important that allo-HLA reactive CD8+ T cells are tested against primary EVT expressing allo-HLA-C, as EVT may have protective mechanisms in place that prevent allo-HLA responses to ensure a successful pregnancy. A recent study described aberrant expression of HLA-DR in syncytiotrophoblasts and syncytiotrophoblast-derived extracellular vesicles (STEVs) in pre-eclampsia but not control placentae, addressing the importance of further examining heterologous immunity of not only decidual CD8+ T cells, but also decidual CD4+ T cells (47).

Not only in the pregnancy setting has HLA-C disparity been described as a possible cause of complication. In transplantation, HLA-C incompatibility has been associated with graft failure after bone marrow transplantation (48). Furthermore, HLA-C mismatches were significantly correlated with acute transplant rejection and increased chronic graft-vs.-host disease (GvHD) after hematopoietic stem cell transplantation (49, 50). Graft loss after solid organ transplantation and GvHD after hematopoietic stem cell transplantation (51) have been associated with heterologous immunity against allo-HLA-A and -B. The proven alloreactivity of virus-specific CD8+ T cells against HLA-C could lead to allo-immune responses and add an additional barrier to tolerance that requires further assessment in transplantation.

In conclusion, alloreactivity against HLA-C occurs and may have pronounced clinical implications in pregnancy, where the only polymorphic allo-HLA antigen expressed by EVT is HLA-C. It remains to be established how often this alloreactivity could lead to the development of pregnancy complications, such as recurrent miscarriages.

## 4. Materials and Methods

### 4.1. Preparation of Responder and Target Cells

Peripheral blood leukocytes were isolated from buffy coats obtained from healthy blood donors after informed consent, at Sanquin Blood Supply, the Netherlands. PBMC were isolated by standard density gradient centrifugation and cryopreserved until use. Single HLA antigen–transfected K562 cells (SALs) were generated as described previously (52). HLA typing was performed by sequence-specific oligonucleotide or sequence-specific primer genotyping at the Department of Immunohematology and Blood Transfusion, LUMC, the Netherlands. Epstein-Barr virus transformed lymphoblastoid cell lines (EBV-LCLs) were generated by incubating PBMC with the supernatant of the EBV-producing marmoset cell line B95.8 for 1.5 h at 37°C, and additional culture in RPMI 1640 Medium (Gibco Life Technologies, Carlsbad, CA) supplemented with 10% Fetal Calf Serum (FCS; Sigma Aldrich, St. Louis, Missouri), Penicillin/Streptavidin (Pen/Strep) and L-glutamine (all from Gibco). The 721.221 cell line expressing HLA-C^*^07:02 was kindly obtained from Professor Anthony W. Purcell (Monash University; cell line originally made by the laboratory of Prof. Andrew Brooks at the University of Melbourne).

Phytohaemagglutinin (PHA) blasts were generated by incubating PBMC for 8 days in RPMI 1640 Medium, Pen/Strep, L-glutamine, 15% human serum (HS, Sanquin, Amsterdam, the Netherlands), IL-2 (60 IU/ml; Novartis, Novartis, Horsham, UK) and PHA (4 μg/ml; Murex Biotech Ltd, Dartford, UK). Human umbilical vein endothelial cells (HUVECs) were cultured in M199 medium supplemented with 10% Newborn calf Serum (NCS), 1% sodium pyruvate, Pen/Strep (all from Gibco), 0,1% β-mercaptoethanol (0.05M, Sigma Aldrich), 1% sodium heparin (400 IE/ml; LUMC, Leiden, the Netherlands), and bovine purine extract (BPE; 100 μl in 20 ml; Invitrogen, Carlsbad, CA).

### 4.2. Generation of Virus-Specific CD8+ T Cell Lines and Clones

PBMC from EBV+, HCMV+, FLU+, and VZV+ blood donors were stained with phycoerythrin (PE)-labeled viral tetramers HCMV HLA-A^*^02:01/NLV, HCMV HLA-B^*^35:01/IPS, EBV HLA-A^*^02:01/GLC, EBV HLA-B^*^08:01/FLR, EBV HLA-B^*^35:01/YPL, FLU HLA-A^*^02:01/GIL, VZV HLA-A^*^02:01/ALW (Protein facility, Department of Immunohematology and Blood Transfusion, LUMC, Leiden, the Netherlands), and an Alexa-647-labeled viral tetramer HCMV pp65 HLA-C^*^06:02/TRA (NIH Tetramer Core Facility, Emory University, Atlanta, GA) Table 1. The HCMV HLA-C^*^07:02/CRV-specific CD8+ T cell clone was generated by CRV peptide stimulation of PBMC from an HCMV+ HLA-C^*^07:02+ donor. Additional staining with conjugated mouse anti-human monoclonal antibodies CD56, CD14, CD4, CD19 (FITC; BD Biosciences, San Jose, CA), CD45 (PE-Cy5; eBioscience, San Diego, CA), and CD8 (Pacific Orange; ThermoFisher, Waltham, MA) was performed. When staining with the HCMV pp65 HLA-C^*^06:02/TRA tetramer, CD158 (KIR2DL1/S1/S3/S5) and CD158b (KIR2DL2/L3) (PE-Cy7; Biolegend, San Diego, CA) were included in the panel to exclude binding of CD8+ T cells to the tetramer through KIR (38). Tetramer-positive CD8+ T cells were purified with FACS sort based on the expression of CD45+CD8+CD19-CD14-CD56-CD4-KIR- tetramer+ cells. CD8+ T cell lines were generated by sorting 10 tetramer-positive cells per round-bottom 96-well and CD8+ T cell clones by sorting 1 tetramer-positive cell per round-bottom 96-well, respectively. After sorting, tetramer-positive CD8+ T cells were expanded in 96-well plates with irradiated PBMC (4,000 Rad) isolated from buffy coats in Iscove's Modified Dulbecco's Medium (IMDM; Lonza, Basel, Switzerland) supplemented with Pen/Strep, L-Glutamine, 5% HS, 5% FCS, PHA (2 μl/ml; Remel, Lenexa, KS), and IL2 (60 IU/ml). TCR Vα and Vβ usage was determined by DNA sequencing using TCR-specific polymerase chain reaction primers (53) followed by use of the BigDye® Terminator V3.1 Cycle Sequencing Kit (Applied Biosystems, Foster City, CA).

### 4.3. Cytokine Production Assay

After 8 days of expansion with allogeneic irradiated PBMC, CD8+ T cell lines and clones (5 x 10^3^) were incubated with EBV-LCLs and PHA blasts (5 x 10^4^) expressing either self-HLA, self-HLA loaded with viral peptide (incubation 30 min at 37°C; washed thrice), or allo-HLA molecules (duplicate; 96-wells) in IMDM supplemented with Pen/Strep, L-Glutamine, 5% HS, 5% FCS, and IL-2 (60 IU /ml). PHA blasts were irradiated (5,000 Rad) before co-culture with T cells. After 24 h at 37°C, supernatants were collected and frozen until further use. IFNγ levels were measured in a standard enzyme-linked immunosorbent assay (ELISA), according to manufacturer's protocol (U-Cytech, Utrecht, the Netherlands). The range of the ELISA standard curve was 5–5120 pg/ml.

### 4.4. Cytotoxicity Assays

After 8 days of expansion with allogeneic irradiated PBMC, serial dilutions of responder CD8+ T cell lines and clones were incubated with^51^Chromium-labeled EBV-LCLs, SALs and/or 721.221 target cells, PHA blasts and HUVECs (responder/stimulator ratio 30:1; 10:1; 1:1; 0.1:1) in round-bottom 96-wells plates for 4 or 20 h at 37°C in IMDM supplemented with Pen/Strep, L-Glutamine, 5% HS, 5% FCS, and IL-2 (60 IU/ml). Where applicable, viral peptide was loaded onto the target cells for 60 min at 37°C, simultaneously with chromium incubation, and washed thrice. In addition, CD8+ T cell lines and clones were incubated with the anti-CD8 blocking antibody FK18 (4.3 μl/ml) for 60 min at 37°C, where after cells were washed twice. Supernatants were harvested for analysis on a gamma-counter (PerkinElmer 2470 Wizard2, Waltham, MA), counts from triplicate wells were averaged, and specific lysis was calculated as follows: (Condition of interest^51^Cr release − Spontaneous^51^Cr release)/(Maximum^51^Cr release − Spontaneous^51^Cr release) x 100. Maximum^51^Cr release of the target cells was determined in PBS 1% Triton X-100 and spontaneous^51^Cr release in medium.

## Ethics Statement

This study was carried out in accordance with the guidelines issued by the Medical Ethics Committee of the Leiden University Medical Center. All subjects gave written informed consent in accordance with the Declaration of Helsinki.

## Author Contributions

AvdZ, EvdM-P, FC, and SH designed the research and wrote the manuscript. AvdZ, EvdM-P and PvM performed the experiments. HvdH and EvdM-P generated the HLA-A and -B-restricted virus-specific T cell lines and clones. JA performed TCR sequencing analyses and DR provided extensive HLA typing.

### Conflict of Interest Statement

The authors declare that the research was conducted in the absence of any commercial or financial relationships that could be construed as a potential conflict of interest.
