# Supplementary material for: Cross-Reactivity of Virus-Specific CD8+ T Cells Against Allogeneic HLA-C: Possible Implications for Pregnancy Outcome
Source: Front Immunol. 2018 Dec 6;9:2880. doi: 10.3389/fimmu.2018.02880 (PMC6291497; doi:10.3389/fimmu.2018.02880)
Supplement: Supplementary file 1 [file Data_Sheet_1.pdf]

Supplementary Material: Cross-reactivity of Virus-specific CD8+ T Cells Against Allogeneic HLA-C: Possible Implications for Pregnancy Outcome

FIGURES

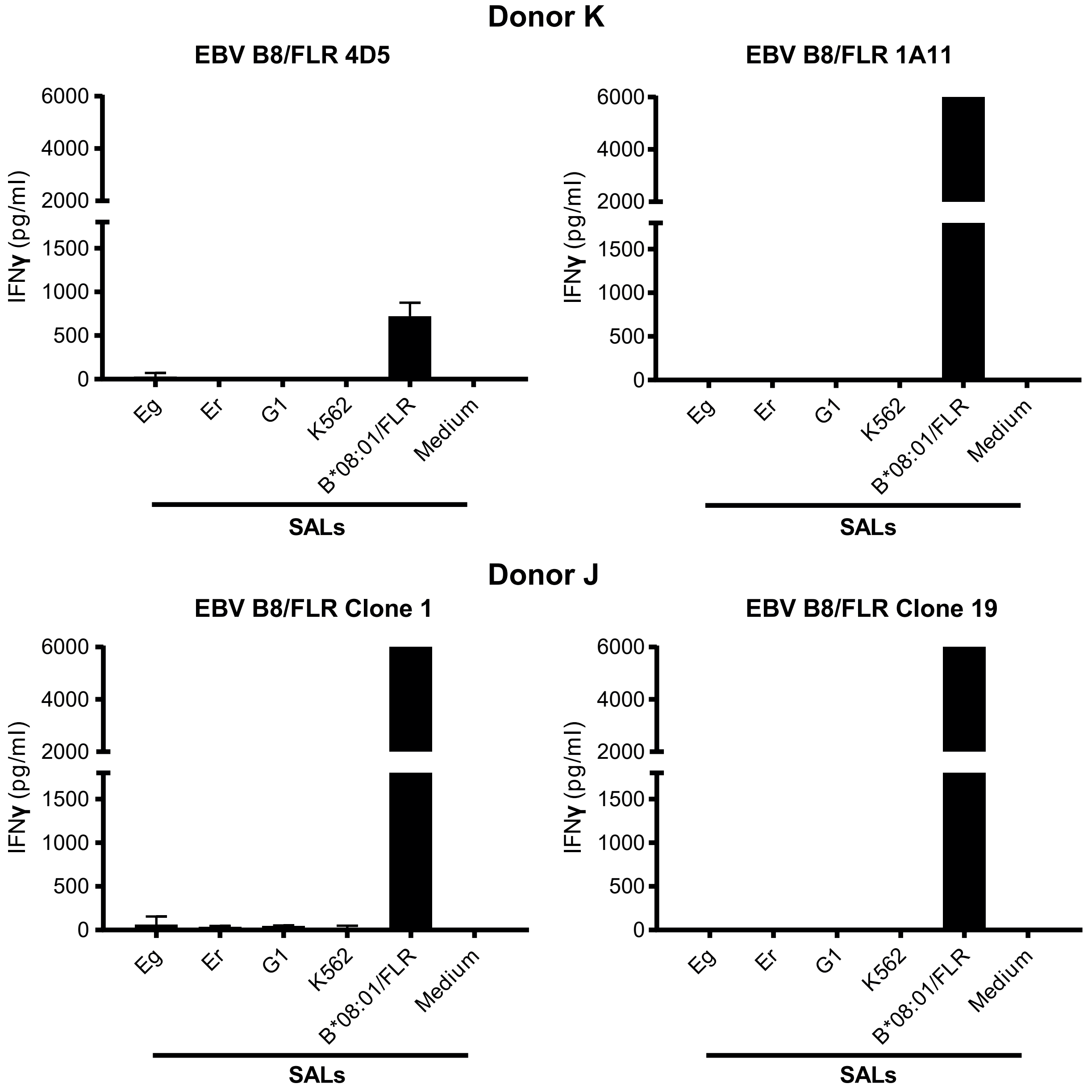

Figure S1. No alloreactivity of EBV B8/FLR T cell lines and clones against HLA-E and -G. An EBV B8/FLR T cell line (1A11) and T cell clones (n=4; 4D5, clone 1 and clone 19 shown) were stimulated with a panel of SALs expressing HLA-E and -G alleles after which IFNγ production was measured. No alloreactivity was observed. The range of the ELISA standard curve: 5 - 5120 pg/ml. Bars represent duplicate values with standard deviation of the mean.

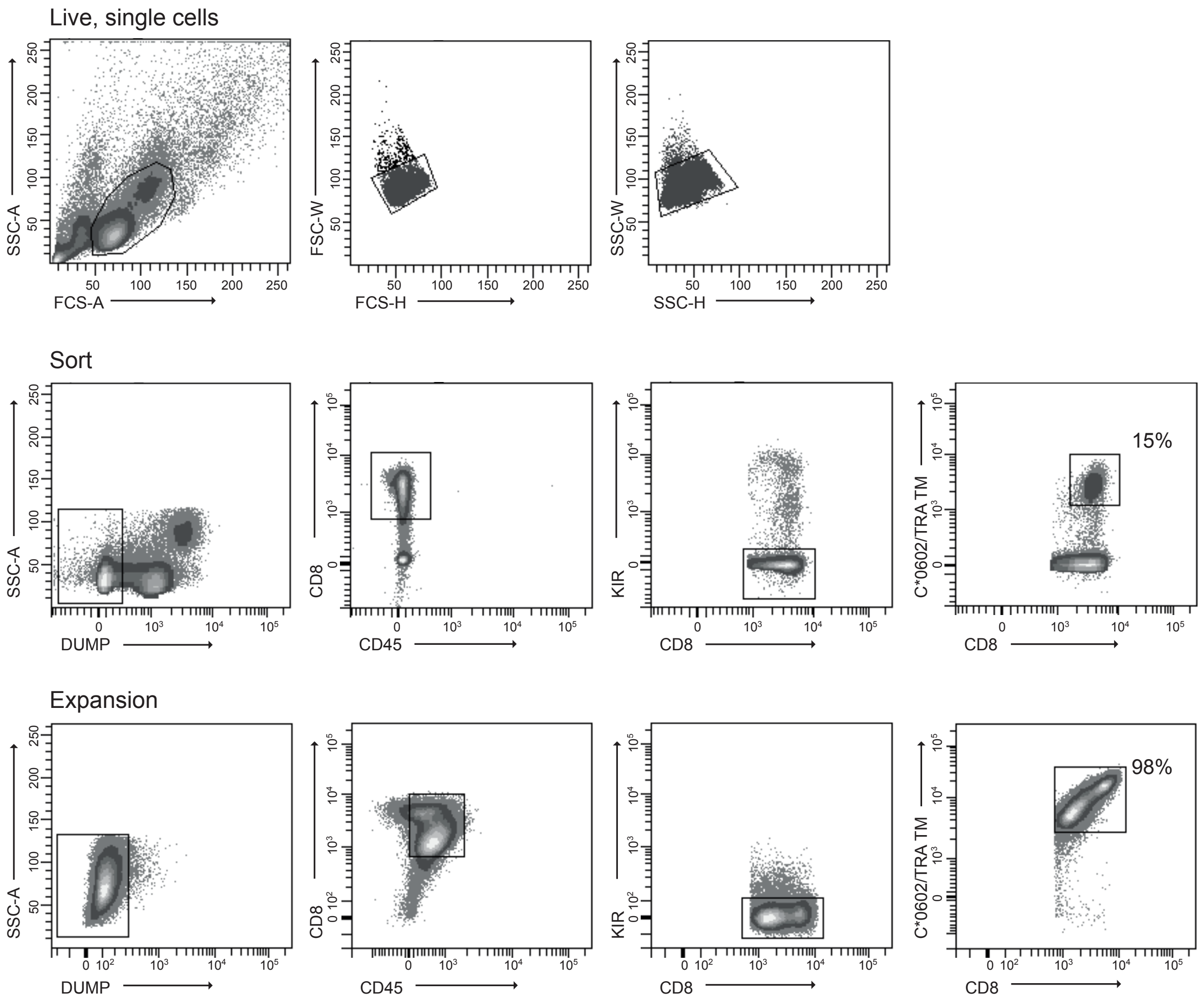

Figure S2. Gating strategy for isolating HLA-C*06:02/TRA-restricted HCMV-specific CD8+ T cells. HCMV HLA-C*06:02/TRA+ CD8+ T cells were identified and sorted by selecting live cells, excluding duplicates (Upper), and selecting DUMP-(CD4-CD14-CD56-~~CD19~~) KIR-tetramer(TM)+ CD8+ T cells (Middle). Sorted tetramer-positive CD8+ T cells were expanded for 2 weeks with irradiated PBMC (Lower).

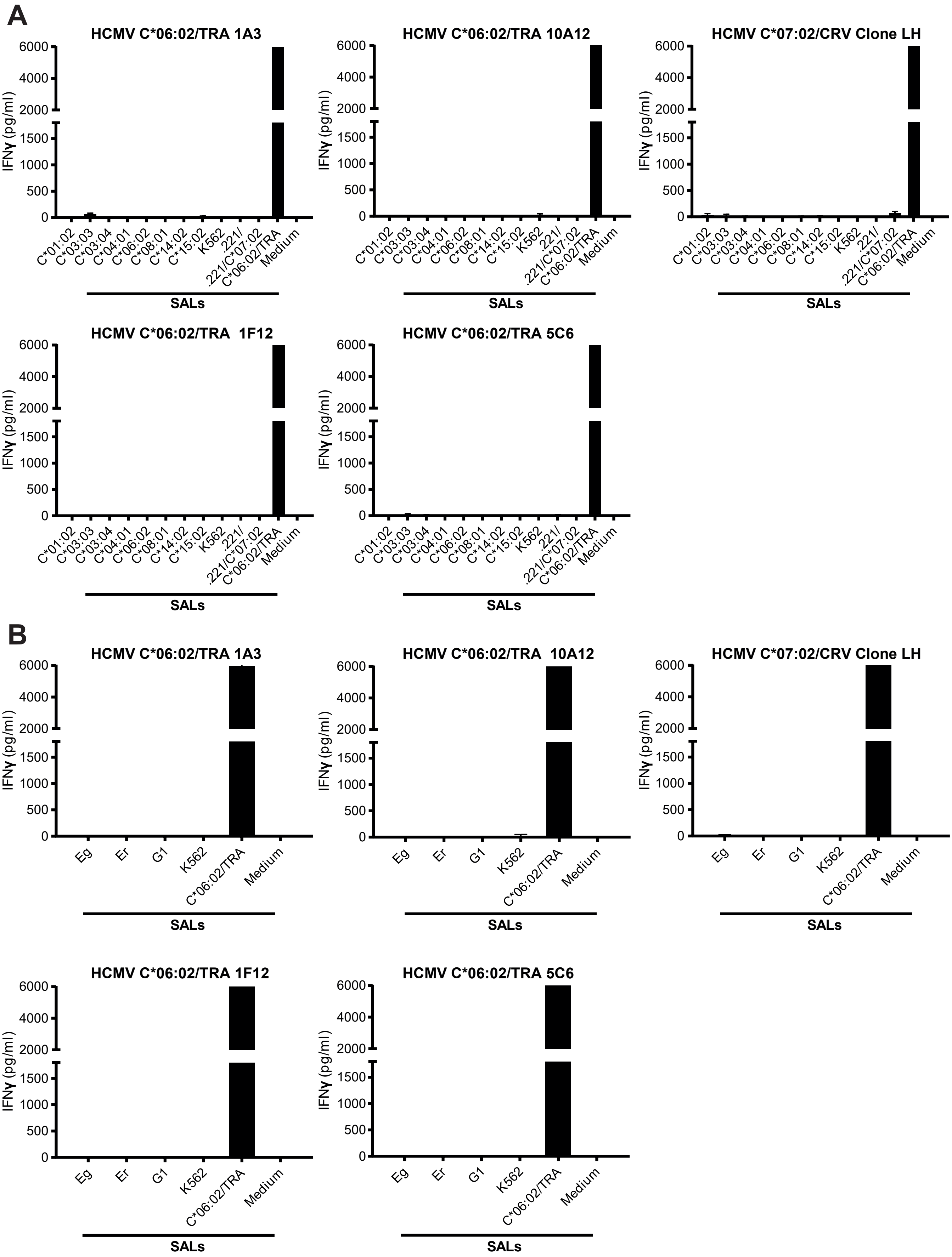

Figure S3. No alloreactivity of HCMV C*06:02/TRA T cell lines and clones, and the HCMV C*07:02/CRV T cell clone against a panel of SALs expressing allogeneic HLA-C, E, and -G. Two HCMV C*06:02/TRA T cell lines (1A3, 10A12) and two T cell clones (1F12, 5C6), and HCMV C*07:02/CRV T cell clone LH were stimulated with a panel of SALs expressing HLA-C alleles (A) and HLA-E and -G alleles (B) after which IFNγ production was measured. No alloreactivity was observed in this setting. The range of the ELISA standard curve: 5 - 5120 pg/ml. Bars represent duplicate values with standard deviation of the mean.

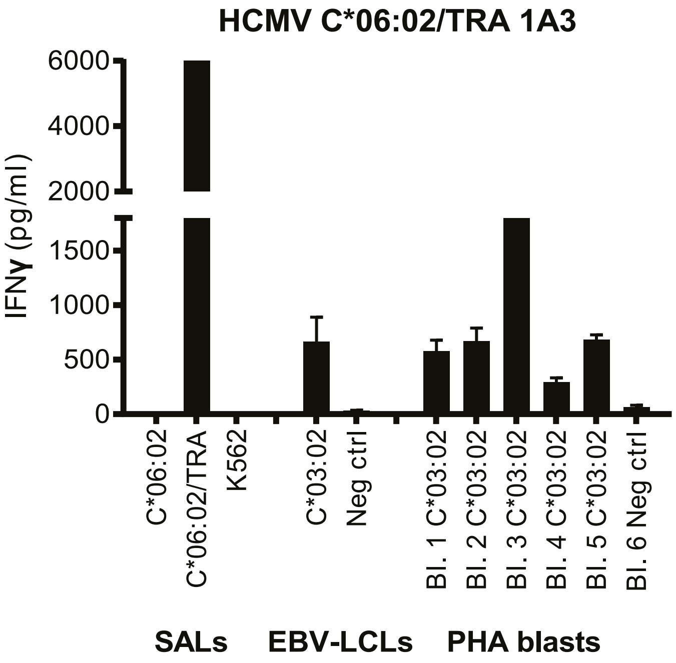

Figure S4. Alloreactivity of the HCMV C*06:02/TRA cell line 1A3 against HLA-C*03:02 expressed on EBV-LCLs and PHA blasts. The HCMV C*06:02/TRA T cell line 1A3 was stimulated with EBV-LCLs and PHA blasts expressing the recognized allo-HLA-C*03:02 allele. SALs expressing HLA-C*06:02 loaded with viral peptide were included as a positive control. The range of the ELISA standard curve: 5 - 5120 pg/ml. Bars represent duplicate values with standard deviation of the mean.
